# Antenatal Corticosteroids: *Primum non nocere*

**DOI:** 10.9745/GHSP-D-18-00461

**Published:** 2018-12-27

**Authors:** Steve Hodgins

**Affiliations:** a Editor-in-Chief, Global Health: Science and Practice Journal, and Associate Professor, School of Public Health, University of Alberta, Edmonton, Alberta, Canada.

## Abstract

Efforts continue—building on work of the UN Commission on Life-Saving Commodities for Women and Children—to expand use of antenatal corticosteroids in low-resource settings. We argue that until more is known on the balance of benefit versus harm, such promotion should be suspended.

See related article by Greensides.

Corticosteroids are a synthetic version of the stress hormone cortisol. As such, they have manifold effects across systems and functions of the body including renal, vascular, endocrine, central nervous system, skin, gut, and—of most relevance for their use for imminent preterm labor—growth and development and immunological function (and, therefore, response to infection).

We have evidence for the protective efficacy of antenatal corticosteroids, dating back to animal studies in the 1960s and clinical trials beginning in the 1970s. Roberts and Dalziel's Cochrane reviews^1^ summarize the findings of these trials (mainly from high-income countries), notably that treatment with antenatal corticosteroids prior to imminent preterm birth, compared with placebo or no treatment, is associated with:

- 32% lower neonatal mortality (relative risk, 0.69; 95% confidence interval, 0.59 to 0.81; N=7188; 22 studies, from the 2017 review), and
- reduced risk of respiratory distress syndrome, intraventricular hemorrhage, necrotising enterocolitis, and systemic newborn infection in the first 48 hours of life.

On the strength of such evidence, corticosteroids have been a mainstay of preventive treatment for preterm birth in high-income countries for decades. Use in low- and middle-income countries (LMICs) has been much less widespread, and evidence much sparser. However, anticipating that such use could have particular benefit in settings with high newborn mortality, there have been prominent efforts at the global level to promote use in LMICs—dating from 2012—reflected in the World Health Organization's (WHO's) *Born Too Soon* report,^2^ the focus on antenatal corticosteroids by the UN Commission on Life-Saving Commodities for Women and Children,^3^ and several prominent papers published at the time.^4^^,^^5^

Corticosteroids have been a mainstay of preventive treatment for preterm birth in high-income countries for decades.

## WORRIES ABOUT EFFICACY AND SAFETY IN LOW- AND MIDDLE-INCOME COUNTRIES

Over the same period as this recent big advocacy push, the Global Network for Women's and Children's Health Research, funded by the National Institute of Child Health and Human Development, was also conducting a large (N=99,742), multi-country, pragmatic trial (called ACT) in Argentina, Guatemala, India, Kenya, Pakistan, and Zambia, encouraging wider use of antenatal corticosteroids for imminent preterm birth, including in peripheral-level health services. The results, first published in October 2014,^6^ came as a shock.^7^ In the intervention arm, neonatal mortality was 12% *higher* than in the comparison arm, corresponding to more than 150 excess deaths, mainly in bigger newborns, presumably most born at or near term. However, even in those in the lowest 5% by birthweight—among whom no overall mortality effect was seen—this may well reflect a mix of individuals benefited and harmed. The clearest evidence of an adverse mortality effect was in the 2 African sites^8^ (although mortality was also elevated in the Indian sites); in addition to higher mortality, these African sites also showed almost a doubling of risk of possible severe bacterial infection among newborns in the intervention arm. Note, also, that stillbirths increased to approximately the same degree as newborn deaths, although evidence^9^ for a causal role of antenatal corticosteroids in risk of stillbirth is less robust.

In the ACT trial conducted in 6 LMICs, use of antenatal corticosteroids was associated with 12% *higher* risk of neonatal mortality.

## RESPONSE TO THE NEW EVIDENCE

In the wake of release of these findings, momentum for expanding use of antenatal corticosteroids in LMICs slowed markedly, though not to a complete halt. The UN Commission on Life-Saving Commodities for Women and Children continued its work^10^—somewhat more cautiously than before—supporting increased use in LMICs. But WHO did issue new, more explicit guidelines in 2015,^12^ specifying the full set of conditions that need to be met for safe use (i.e., for the probability of benefit to be greater than the probability of harm), notably:

- Accurate gestational age dating and competent assessment determining imminent preterm birth (i.e., within 1–7 days)
- No evidence of maternal infection
- Adequate care at childbirth and for preterm newborns, including thermal care, nutrition/fluids, infection treatment, and care for respiratory complications (including safe oxygen use)

## HOW IMPORTANT ARE THESE REQUIREMENTS?

### Ensuring Accurate Selection and Timing Based on Accurate Estimation of Gestational Age

From the results of the ACT trial,^6^ excess mortality was seen only among those in the highest 75% by birthweight. This group would have included few newborns in the target gestational age of <34 weeks, but mainly a mix of mistimed administration and pregnancies exposed to antenatal corticosteroids at an appropriate gestational age but continuing on to term or near term before delivering. There had already been an indication in secondary analyses done by Roberts^1^ that for this later gestational age group, the probability of harm could exceed probability of benefit. The recently published, U.S.-based Antenatal Late Preterm Steroids (ALPS) trial,^13^ on antenatal corticosteroids for late preterm gestation (34 weeks 0 days, to 36 weeks 5 days), documented a reduced risk of respiratory complications of prematurity. However, it found no impact on mortality and an increased risk of neonatal hypoglycemia (and evidence from elsewhere suggests this effect may be more severe for mothers of low body mass index^14^). The relevance of these findings for LMIC settings is unclear. Although mortality risk is markedly lower in this older gestational age group than among those <34 weeks at birth, they are far more numerous and therefore still account for a large number of deaths in LMICs. It certainly appears that in the older gestational age group, in LMIC settings similar to the ACT trial, probability of harm from antenatal corticosteroids exposure exceeds probability of benefit. So, accuracy of both gestational age dating and assessment of imminence of birth appears to be quite important.

Accuracy of gestational age dating and assessment of imminence of birth appears to be quite important for the safe use of antenatal corticosteroids.

### Challenges in Assessing Gestational Age Especially Late in Pregnancy

In the ACT trial settings, obstetrical ultrasound was generally unavailable. Investigators made a serious effort to ensure that participating health workers had training and equipment to enable them to competently estimate gestational age based on last normal menstrual period and fundal height, within the limitations of the method. However, numerous studies^15^ have found that such clinical assessment of gestational age is associated with greater error than first trimester ultrasound.

The most accurate ultrasound dating is by first trimester crown-rump length (accurate to within +/− 3 to 8 days).^16^ In the second and third trimester, the most accurate estimates are by an index of several measurements.^15^ Currently, the standard is based on femur length and head circumference. According to a recent study by Papageorghiou using this index,^17^ accuracy in the third trimester can be +/− 14 days or slightly more.

So, competently done clinical assessment using last normal menstrual period and fundal height can yield accuracy within about 2 weeks, if done in the first trimester, but accuracy declines with gestational age. Competently done ultrasound in the first trimester, using crown-rump length, gives the greatest accuracy. Ultrasound done later in pregnancy is less accurate, with imprecision of 14 days or more in the third trimester.

In principle, one way of improving targeting of antenatal corticosteroids would be to make competent obstetrical ultrasound more widely available in LMICs. However, effectiveness and feasibility of such a strategy was tested in the FirstLook study, which documented that although it was possible to get good-quality assessments, there were significant challenges even with robust technical support.^18^

In summary, ensuring appropriate timing of administration—which depends on accurate gestational age estimation—matters. But in many LMIC settings, it's highly problematic to get accurate dating.

In many LMICs, it's highly problematic to get accurate gestational age dating.

### Ruling Out Maternal Infection

A second condition specified in the new WHO recommendations is that maternal infection be ruled out. Given evidence from the ACT trial of higher rates of maternal infection and potentially severe infection in the newborns in the intervention arm, it would appear appropriate to insist on carefully ruling out maternal infection before administering antenatal corticosteroids. However, it's not clear that had such provisions been more robust in the ACT trial, they would have been sufficient to ensure safe use.

### Standard of Care for Preterm Newborns

The WHO guidelines also specify—as a requirement for safe use—that the service delivery environment be capable of providing adequate care for preterm newborns, including appropriate thermal care, managing feeding and fluids, and care of respiratory complications (including safe oxygen use). The fact that a health facility is considered capable of providing emergency obstetrical care (including cesarean delivery) is no guarantee that it also has the capability of providing adequate care for preterm newborns and their complications. Furthermore, while we can expect that having such capability would contribute to reducing mortality risk, this is only one of a full set of conditions to be met for safe antenatal corticosteroids use. In settings where the other conditions are not met (e.g., accurate gestational age dating using first or second trimester ultrasound), even when antenatal corticosteroids are used in highly functional tertiary-level health facilities, one cannot necessarily be confident that the probability of benefit would exceed probability of harm.

### Other Potentially Relevant Contextual Differences Between High-Income and Low- and Middle-Income Countries

Although not mentioned in the WHO guidelines, other authors^8^ have pointed out that not only does the service delivery environment differ between high-income countries and LMICs, but there may also be relevant epidemiologic differences that can influence the balance of benefit versus harm (and, indeed, there may be important differences *between* LMIC settings, for example, between South Asia and sub-Saharan Africa). Notably, there may be relevant differences in microbiological exposures and nutritional factors and associated responses in the mother and fetus. More needs to be learned before we can be confident of the results to be expected using this treatment across varied epidemiologic settings.

## ADDRESSING UNCERTAINTIES

Several authors have recently pointed to uncertainties with regard to the balance of benefit versus risk in LMICs. Jobe and Goldenberg^19^ comment, on antenatal corticosteroids: “an effective therapy in high-resource environments may be ineffective or harmful in low-resource environments.” In the recently updated Cochrane review by Roberts et al.,^1^ the authors conclude that it would be particularly relevant to explore effectiveness versus possible harms in low-resource settings, using adequately powered prospective trials. And this call is echoed in Vogel's report^20^ on the conclusions of expert meetings convened by WHO in 2015 and 2016. In response to such calls for further trials (including in older gestational-age pregnancies), funds have been committed for 2 new trials in LMICs to be implemented under WHO: ACTION I (testing antenatal corticosteroids use <34 weeks gestation)^21^ and ACTION II (34 to 36 weeks).^22^

## AWAITING NEW EVIDENCE, WHAT SHOULD BE OUR STANCE IN LMIC SETTINGS?

From what was documented in the article in this issue of GHSP by Greensides et al.,^11^ it is pretty clear that conditions for safe use—as specified in the current WHO recommendations—are ***not*** consistently being met in the countries surveyed. It is certainly plausible that current use of antenatal corticosteroids in these settings could be resulting in more deaths caused than prevented.

In our view, global leaders in maternal and newborn health need to communicate more clearly: if we cannot be confident that all essential conditions for safe use are met, antenatal corticosteroids may *increase* rather than reduce risk of newborn death. On the principle of *primum non nocere*—first, do no harm—we should not promote use of antenatal corticosteroids in LMIC settings unless or until we have robust evidence that expectation of benefit exceeds expectation of harm.

*Primum non nocere*: Until we have better evidence on the balance of effectiveness and safety, we should not promote antenatal corticosteroids in LMICs.

## Notes

**Funding:** None.

**First Published Online:** December 20, 2018

## References

[1] Roberts D Dalziel S. Antenatal corticosteroids for accelerating fetal lung maturation for women at risk of preterm birth. Cochrane Database Syst Rev. 2006;(3):CD004454. Update in: Cochrane Database Syst Rev. 2017;(3):CD004454. 10.1002/14651858.CD004454.pub2. 16856047

[2] March of Dimes; Partnership for Maternal, Newborn, and Child Health; Save the Children; World Health Organization (WHO). Born Too Soon: The Global Action Report on Preterm Birth. Geneva: WHO; 2012. https://www.healthynewbornnetwork.org/hnn-content/uploads/BornTooSoon-Report-April2012.pdf. Accessed November 29, 2018.

[3] United Nations, Every Woman Every Child. UN Commission on Life-Saving Commodities for Women and Children: Commissioners' Report September 2012. New York: United Nations; 2012. https://www.unicef.org/spanish/media/files/UN_Commission_Report_September_2012_Final.pdf. Accessed November 29, 2018.

[4] Lawn JE Kinney MV Belizan JM,; Born Too Soon Preterm Birth Action Group. Born too soon: accelerating actions for prevention and care of 15 million newborns born too soon. Reprod Health. 2013;10(suppl 1):S6. 10.1186/1742-4755-10-S1-S6. 24625252 PMC3828574

[5] Lawn JE Segre J Barker P,. Antenatal corticosteroids to reduce preterm deaths in low-income settings. Lancet Glob Health. 2014;2(8):e446. 10.1016/S2214-109X(14)70263-3. 25103512

[6] Althabe F Belizán JM McClure EM,. A population-based, multifaceted strategy to implement antenatal corticosteroid treatment versus standard care for the reduction of neonatal mortality due to preterm birth in low-income and middle-income countries: the ACT cluster-randomised trial. Lancet. 2015;385(9968):629–639. 10.1016/S0140-6736(14)61651-2. 25458726 PMC4420619

[7] Hodgins S. Caution on corticosteroids for preterm delivery: learning from missteps. Glob Health Sci Pract. 2014;2(4):371–3. 10.9745/GHSP-D-14-00197. 25611469 PMC4307851

[8] Klein K McClure EM Colaci D,. The Antenatal Corticosteroids Trial (ACT): a secondary analysis to explore site differences in a multi-country trial. Reprod Health. 2016;13(1):64. 10.1186/s12978-016-0179-z. 27221319 PMC4878061

[9] Goldenberg RL Thorsten VR Althabe F,. The global network antenatal corticosteroids trial: impact on stillbirth. Reprod Health. 2016;13(1):68. 10.1186/s12978-016-0174-4. 27255082 PMC4891888

[10] Pronyk PM Nemser B Maliqi B,; UNCoLSC Technical Resource Teams; UN Agency Leads; UNCoLSC Monitoring and Evaluation Advisory Group. The UN Commission on Life Saving Commodities 3 years on: global progress update and results of a multicountry assessment. Lancet Glob Health. 2016;4(4):e276–86. 10.1016/S2214-109X(16)00046-2. 27013314

[11] Greensides D Robb-McCord J Noriega A Litch JA. Antenatal corticosteroids for women at risk of imminent preterm birth in 7 sub-Saharan African countries: a policy and implementation landscape analysis. Glob Health Sci Pract. 2018;6(4). 10.9745/GHSP-D-18-00171 PMC6370350 30573455

[12] World Health Organization (WHO). WHO Recommendations on Interventions to Improve Preterm Birth Outcomes. Geneva: WHO; 2015. https://www.who.int/reproductivehealth/publications/maternal_perinatal_health/preterm-birth-guideline/en/. Accessed November 29, 2018. 26447264

[13] Gyamfi-Bannerman C Thom EA Blackwell SC,; NICHD Maternal-Fetal Medicine Unis Network. Antenatal betamethasone for women at risk for late preterm delivery. N Engl J Med. 2016;374(14):1311–1320. 10.1056/NEJMoa1516783. 26842679 PMC4823164

[14] Jolley JA Rajan PV Petersen R Fong A Wing DA. Effect of antenatal betamethasone on blood glucose levels in women with and without diabetes. Diabetes Res Clin Pract. 2016;118:98–104. 10.1016/j.diabres.2016.06.005. 27351800

[15] Hadlock FP Harrist RB Shah YP,. Estimating fetal age using multiple parameters: a prospective evaluation in a racially mixed population. Am J Obstet Gynecol. 1987;156(4):955–957. 3578406 10.1016/0002-9378(87)90365-6

[16] Butt K Lim K. Determination of gestational age by ultrasound. J Obstet Gynaecol Can. 2014;36(2):171–181. 10.1016/S1701-2163(15)30664-2 24518917

[17] Papageorghiou AT Kemp B Stones W,; International Fetal and Newborn Growth Consortium for the 21st Century (INTERGROWTH-21st). Ultrasound-based gestational-age estimation in late pregnancy. Ultrasound Obstet Gynecol. 2016;48(6):719–726. 10.1002/uog.15894. 26924421 PMC6680349

[18] Swanson D Lokangaka A Bauserman M,. Challenges of implementing antenatal ultrasound screening in a rural study site: a case study from the Democratic Republic of the Congo. Glob Health Sci Pract. 2017;5(2):315–324. 10.9745/GHSP-D-16-00191. 28655805 PMC5487092

[19] Jobe AH Goldenberg RL. Antenatal corticosteroids: an assessment of anticipated benefits and potential risks. Am J Obstet Gynecol. 2018;219(1):62–74. 10.1016/j.ajog.2018.04.007. 29630886

[20] Vogel JP Oladapo OT Pileggi-Castro C,. Antenatal corticosteroids for women at risk of imminent preterm birth in low-resource countries: the case for equipoise and the need for efficacy trials. BMJ Glob Health. 2017;2(3):e000398. 10.1136/bmjgh-2017-000398. 29082019 PMC5656119

[21] UNDP/UNFPA/UNICEF/WHO/World Bank Special Programme of Research, Development, and Research Training in Human Reproduction (HRP). The WHO ACTION-I (Antenatal Corticosteroids for Improving Outcomes in Preterm Newborns) Trial. http://www.who.int/reproductivehealth/projects/ACTION-I.pdf. Accessed November 29, 2018.

[22] UNDP/UNFPA/UNICEF/WHO/World Bank Special Programme of Research, Development, and Research Training in Human Reproduction (HRP). The WHO ACTION-II (Antenatal Corticosteroids for Improving Outcomes in Preterm Newborns) Trial. http://www.who.int/reproductivehealth/projects/ACTION-II.pdf. Accessed November 29, 2018.

